# The First Report of the Prion Protein Gene (*PRNP*) Sequence in Pekin Ducks (*Anas platyrhynchos domestica*): The Potential Prion Disease Susceptibility in Ducks

**DOI:** 10.3390/genes12020193

**Published:** 2021-01-28

**Authors:** Min-Ju Jeong, Yong-Chan Kim, Byung-Hoon Jeong

**Affiliations:** 1 Korea Zoonosis Research Institute, Jeonbuk National University, Iksan, Jeonbuk 54531, Korea; minju5149@jbnu.ac.kr (M.-J.J.); kych@jbnu.ac.kr (Y.-C.K.); 2 Department of Bioactive Material Sciences, Jeonbuk National University, Jeonju, Jeonbuk 54896, Korea

**Keywords:** duck, prion, prion protein gene, *PRNP*, prion protein, PrP, pekin duck

## Abstract

Pathogenic prion protein (PrP^Sc^), converted from normal prion protein (PrP^C^), causes prion disease. Although prion disease has been reported in several mammalian species, chickens are known to show strong resistance to prion diseases. In addition to chickens, the domestic duck occupies a large proportion in the poultry industry and may be regarded as a potential resistant host against prion disease. However, the DNA sequence of the prion protein gene (*PRNP*) has not been reported in domestic ducks. Here, we performed amplicon sequencing targeting the duck *PRNP* gene with the genomic DNA of Pekin ducks. In addition, we aligned the PrP sequence of the Pekin duck with that of various species using ClustalW2 and carried out phylogenetic analysis using Molecular Evolutionary Genetics Analysis X (MEGA X). We also constructed the structural modeling of the tertiary and secondary structures in avian PrP using SWISS-MODEL. Last, we investigated the aggregation propensity on Pekin duck PrP using AMYCO. We first reported the DNA sequence of the *PRNP* gene in Pekin ducks and found that the PrP sequence of Pekin ducks is more similar to that of geese than to that of chickens and mallards (wild ducks). Interestingly, Pekin duck PrP showed a high proportion of β-sheets compared to that of chicken PrP, and a high aggregation propensity compared to that of avian PrPs. However, Pekin duck PrP with substitutions of chicken-specific amino acids showed reduced aggregation propensities. To the best of our knowledge, this is the first report on the genetic characteristics of the *PRNP* sequence in Pekin ducks.

## 1. Introduction

Prion diseases are lethal neurodegenerative diseases and are shown in wild host ranges among mammals [1,2,3,4,5,6,7,8,9,10]. Prion protein (PrP) acts as an important molecule in the pathogenesis of prion disease [11]. The natural cellular form of PrP (PrP^C^) is converted to a pathogenic unnatural form of PrP (PrP^Sc^) by protein misfolding [1,12]. PrP^Sc^ is characterized by β-sheet-rich aggregates that accumulate in the brain, resulting in prion disease [1,12].

Recent reports have suggested that the specific PrP sequences of dogs, horses and chickens, which are prion disease-resistant species, may contribute to resistance against prion diseases [13,14,15,16,17]. In dogs, a dog-specific amino acid (an aspartic acid (Asp) at codon 163) seems likely to be associated with the resistance of prion disease. A previous study reported that a substitution at codon 158 (mouse PrP numbering corresponds to codon 163 of canine PrP) into Asp in mouse PrP resulted in an additional salt bridge in a tertiary structure model [18]. The additional salt bridge between Arg135 and Asp158 may contribute to the structural stability of PrP and the resistance of conversion into a misfolded protein by protein misfolding cyclic amplification (PMCA). In addition, transgenic mice carrying mouse PrP with the dog-specific amino acid at the codon 158 on the Asp allele showed a prolonged incubation period after infection with various prion strains [19]. In horses, the horse-specific Asp at codon 167, which is located within the β2–α2 loop, was confirmed to contribute to the structural stability of equine PrP [20,21]. The β2–α2 loop with Asp at codon 167 may allow the maintenance of a well-defined PrP structure and contribute to low susceptibility to prion disease in horses [22,23].

The chicken PrP showed an interspecific conserved PrP structure, including a signal peptide, tandem amino acid repeat, hydrophobic core, glycosylation site and globular domain [24,25]. Furthermore, chicken PrP is comprised of three α-helices structures and small anti-parallel β-sheet structures in the C-terminal globular domain, which showed a tertiary structure similar to that of mammals [25,26]. However, despite many similarities with mammalian PrPs, prion disease has not been reported in chickens during the bovine spongiform encephalopathy (BSE) outbreak in the UK [27]. Although global meat consumption of chicken (36.78%) was higher than that of beef (20.69%) and sheep (4.56%), it is noteworthy that prion diseases have not been reported in chickens (UN Food and Agricultural Organization, 2018). Furthermore, a BSE infection experiment by parenteral and oral inoculations in chickens showed that these chickens failed to develop prion disease [28].

Although ducks have accounted for a small portion (1.29%) of total meat consumption, ducks showed a major consumption distribution in the poultry industry (UN Food and Agricultural Organization, 2018), however, as with chickens, prion disease has not been reported in ducks. Thus, studies on prion disease in ducks are necessary to discover characteristics of the PrP sequence associated with resistance to prion diseases; however, even the DNA sequence of the prion protein gene (*PRNP*), which encodes PrP, in domestic Pekin ducks has not been reported thus far. Pekin ducks were domesticated from wild mallard ducks in Southeast Asia and bred into various breeds around world, including American and German subtypes [29,30]. In Korea, most of the Pekin duck breed has been imported in suntypes from England, France, and Germany [31].

The purpose of this study was to identify the DNA sequence of the *PRNP* gene in domestic Pekin ducks and analyze the genetic characteristics of the *PRNP* gene in domestic Pekin ducks compared to those in various species. We amplified the *PRNP* DNA sequence of domestic Pekin ducks and aligned the chicken, wild duck and domestic Pekin duck *PRNP* gene sequences. We also compared the amino acid sequence of PrP and tandem repeats among 10 species and performed phylogenetic analysis using Molecular Evolutionary Genetics Analysis (MEGA) X. In addition, we predicted the tertiary and secondary structures of avian PrP using SWISS-MODEL and Swiss PDB Viewer. Last, we estimated the aggregation propensities into amyloid structures in avian PrP and Pekin duck PrP substituted with chicken-specific amino acids using AMYCO (combined AMYloid and Composition based prediction of the prion-like aggregation propensity).

## 2. Materials and Methods

### 2.1. Samples

Four samples of Pekin ducks were provided by a slaughterhouse in the Republic of Korea. The Labopass Tissue Genomic DNA Isolation Kit (Cosmo Genetech Co., Ltd., Seoul, Korea) was used to isolate genomic DNA from 20 mg peripheral tissue following the manufacturer’s protocols. The overall experimental protocols were approved by the Institutional Animal Care and Use Committee of Jeonbuk National University (CBNU 2017-0030). All experiments with the Pekin duck samples were performed in accordance with the Korea Experimental Animal Protection Act.

### 2.2. Primer Design and Amplification of the Duck PRNP Gene

Based on the mallard *PRNP* sequence (*Anas platyrhynchos*) registered in GenBank at the National Center for Biotechnology Information (NCBI) (Gene ID: AF283319.1), a set of primers was designed and used to amplify the coding region of the Pekin duck *PRNP* gene by polymerase chain reaction (PCR). The gene-specific forward and reverse primers were as follows: DUCK_PRNP-F (5’-TGGTGCAGACAACAGCTGGG-3’) and DUCK_PRNP-R (5’-TGGGCTCAGGGACACGAAGA-3’). The PCR reaction, amplicon sequencing and genotyping were carried out as described in a previous study [17].

### 2.3. Multiple Sequence Alignments and Phylogenetic Analyses

The amino acid sequences of PrP, including humans (*Homo sapiens*, NP_001073590.1), chimpanzee (*Pan troglodytes*, NC_036899.1), macaque (*Macaca fascicularis*, NC_022281.1), cow (*Bos taurus*, NC_037340.1), sheep (*Ovis aries*, NP_001009481.1), goat (*Capra hircus*, NP_001301176.1), dog (*Canis lupus familiaris*, XP_005634748.1), chicken (*Gallus gallus*, NC_006109), mallard (*Anas platyrhynchos*, AF283319.1), and goose (*Anser cygnoides domesticus*, NW_013185922), but not Pekin duck (in this study), were acquired from GenBank for multiple sequence alignments and phylogenetic analyses. The PrP sequences were aligned using ClustalW2 (https://www.ebi.ac.uk/Tools/msa/clustalo/). A phylogenetic tree was constructed by MEGA X using the neighbor-joining method [32]. The bootstrap test from 3000 replicates was applied to estimate the confidence level of the branching patterns of the neighbor-joining tree. The tree is drawn to scale, and branch lengths of equal units as those of the evolutionary distances were used to deduce the phylogenetic tree. The evolutionary distances were calculated using the Poisson correction method and are expressed as the number of amino acid substitutions per site.

### 2.4. Modeling of the Tertiary Structure of Avian PrP

Structure modeling of PrPs was carried out using the structural bioinformatics tool, SWISS-MODEL (https://swissmodel.expasy.org). SWISS-MODEL generates a tertiary structure of a target sequence by searching an evolutionarily related protein structure in the SWISS-MODEL Template Library (SMTL) and deducing the tertiary structure from the NMR structure, which serves as the template. To select a tertiary structure from the derived candidate models, the criteria were as follows: GMQE (Global Model Quality Estimation), QMEAN (Qualitative Model Energy Analysis), and sequence homology. GMQE is used for estimating the quality of the tertiary structure model, by means of the target–template alignment and the template-searching technique, in which higher scores signify a higher reliability of the corresponding model. QMEAN is an estimate of the “degree of nativeness” between the structural features in the observed model and the experimental model, with its scores of −4.0 or below indicating low quality. The NMR structure of chicken PrP (PDB ID: 1U3M) was obtained from the RCSB protein data bank (PDB) (https://www.rcsb.org/). The visualization of the protein structure was carried out with Swiss PDB Viewer 4.1 software.

### 2.5. Prediction of the Aggregation Propensity of Avian PrP

The AMYCO (combined AMYloid and Composition based prediction of the prion-like aggregation propensity) webserver is an application of in silico analysis that predicts the propensity to aggregate into amyloid structure (http://bioinf.uab.cat/amyco/). The AMYCO result provides the visualized AMYCO scores.

## 3. Results

### 3.1. Identification of the PRNP Sequence in Pekin Ducks

To date, the DNA sequence of the *PRNP* gene in Pekin ducks (*Anas platyrhynchos domesticus*, domestic ducks) has not been reported. Thus, to identify the *PRNP* DNA sequence of the Pekin ducks, we designed a pair of primers based on the *PRNP* DNA sequence of the mallard (*Anas platyrhynchos*, wild ducks) registered in GenBank at the NCBI (Gene ID: AF283319.1). PCR was performed with tissue-derived genomic DNA from Pekin ducks. The 894 bp amplicon was confirmed by agarose gel electrophoresis (data not shown). Using this amplicon, we performed direct sequencing analysis and identified the *PRNP* DNA sequence of Pekin ducks for the first time.

**Figure 1:**
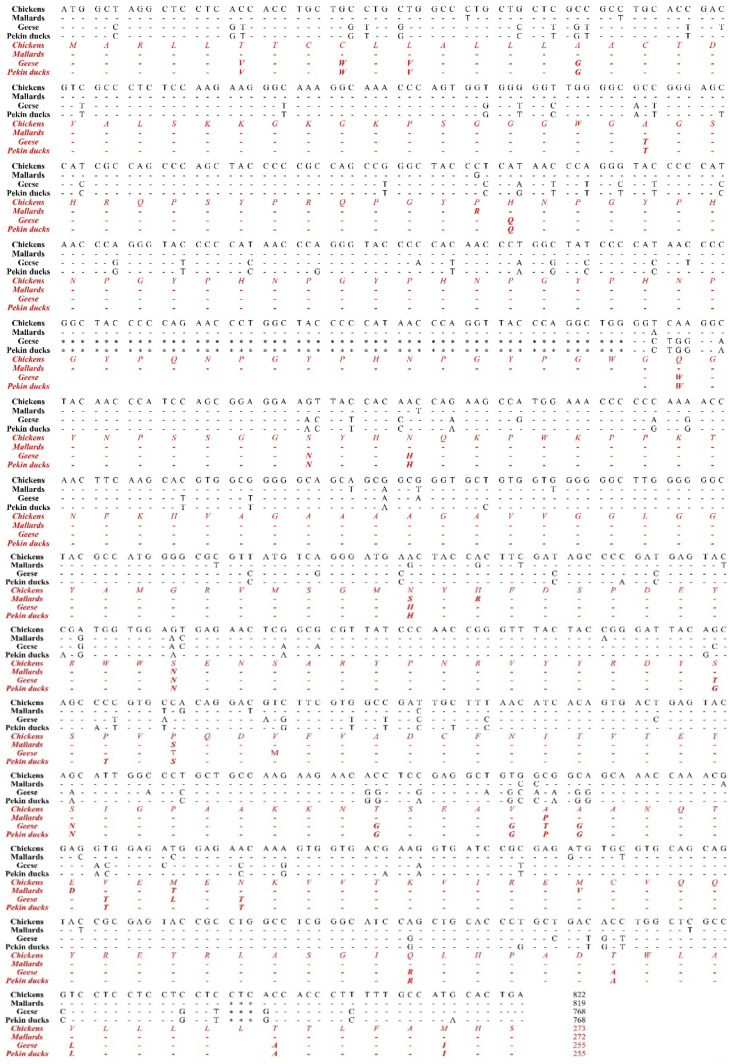
The comparison of the ORF of the *PRNP* gene was performed in chickens, mallards, geese, and Pekin ducks. The black text indicates nucleotide sequences and the red and italic texts indicate amino acid sequences. -, identical nucleotide or amino acid to chicken; *, nucleotide deletion; $, stop codon.

Next, to investigate differences of the open reading frame (ORF) in the *PRNP* gene among birds, we aligned the DNA and amino acid sequences of the *PRNP* gene in chickens, mallards, geese and Pekin ducks to find differences among the sequences (Figure 1). The lengths of the ORF of the *PRNP* gene were different among chickens, mallards, geese and Pekin ducks. In brief, the chicken has the longest ORF with 822 bp (273 aa), followed by that of the mallard with 819 bp (272 aa). Notably, Pekin ducks and geese have the shortest ORF of 768 bp (255 aa). Compared to the PrP amino acid sequence of chickens and mallards, that of Pekin ducks and geese showed a deletion of 51 nucleotides (17 aa: GYPQNPGYPHNPGYPGW) between codons 80 and 81 within the tandem repeat region based on the amino acid sequence of the chickens and mallards. In addition, the chicken PrP amino acid sequence has a 3-bp insertion (CTC, leucine) between codons 248 and 249 near the stop codon based on the amino acid sequence of the Pekin duck compared to that of Pekin ducks, geese and mallards. Since chickens are prion disease-resistant animals, the sequence difference between chickens and Pekin ducks is very important because it may be associated with the susceptibility of prion disease. Overall, we found 155 nucleotide mismatches (20.2% difference) in the *PRNP* gene and 44 different amino acids in the PrP protein between chickens and Pekin ducks. In addition, we found 168 mismatches (21.9% difference) in the *PRNP* gene and 45 different amino acids in the PrP protein between mallard and Pekin ducks. Furthermore, we observed 32 mismatches (4.2% difference) in the *PRNP* gene and 6 different amino acids in the PrP protein between geese and Pekin ducks. The homology of the PrP sequence of Pekin duck was the highest with that of geese (97.3%), followed by that of chickens (82.7%) and mallards (82.4%).

### 3.2. Sequence Alignments and Phylogenetic Analyses of PrP in Several Species

To investigate the sequence homology of PrP between the Pekin duck and several species, we performed multiple sequence alignment in 10 species using ClustalW2 (Figure S1A). We found two Pekin duck-specific amino acids, Gly at codon 163 and Thr at codon 165. The PrP sequence of all mammals showed low homology (less than 40%) compared to that of Pekin ducks, and a dog-specific amino acid (at codon 163), which is associated with the resistance of prion disease, was not observed in all kinds of birds. In addition, we compared tandem repeat regions within the PrP of mammals and birds (Figure S1B). The tandem repeat domain of birds was significantly different from that of mammals (mammals: 5-6 octapeptides, birds: 5-8 hexapeptides). 

Furthermore, we constructed a phylogenetic tree using the neighbor-joining method to evaluate evolutionary relationships of the PrP amino acid sequence of Pekin ducks. In Figure S2, we observed two distinct lineages (avian and mammalian), as expected. The tree illustrates that avian PrP sequences in one group, while mammalian PrP sequences are in another group, which consists of subgroups including primates and ruminants. Interestingly, Pekin ducks showed a closer evolutionary relationship with geese than with mallards (wild ducks).

### 3.3. Analysis of the Tertiary and Secondary Structures of the Pekin Duck PrP

**Figure 2:**
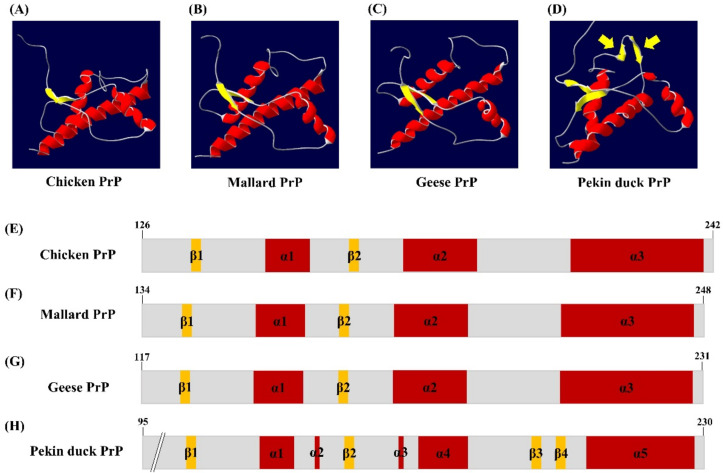
The tertiary and secondary structure of avian prion proteins (PrPs). The diagram of the tertiary structure model and schematic representation of the secondary structure of avian PrPs was constructed by SWISS-MODEL and Swiss PDB Viewer. (**A**) The tertiary structure of the chicken PrP. (**B**) The tertiary structure of the mallard PrP. (**C**) The tertiary structure of the goose PrP. (**D**) The tertiary structure of the Pekin duck PrP. The yellow arrows indicate additional β-sheet structures. (**E**) The secondary structure of the chicken PrP. (**F**) The secondary structure of the mallard PrP. (**G**) The secondary structure of the goose PrP. (**H**) The secondary structure of the Pekin duck PrP. The colors highlight α-helices (red), β-sheets (yellow) and coils (gray).

We analyzed the tertiary structure of the newly discovered PrP sequence of Pekin ducks using two programs, SWISS-MODEL and Swiss-PDB Viewer. The tertiary structure of the PrP in Pekin ducks was constructed to a model according to modeling evaluation values (GMQE and QMEAN) and sequence identity (Figure 2D). Interestingly, the tertiary structure of PrP of Pekin ducks obviously showed a structural difference compared with that of PrP of chickens, mallards and geese (Figure 2A–C). As shown in Figure 2D, these structural differences were confirmed to shorten the α-helices and additional β-sheet structures between the α4 helix and α5 helix. Furthermore, we investigated the secondary structure among avian PrPs. The secondary structure of chickens, mallards and geese showed three α-helices structures and two small anti-parallel β-sheet structures (Figure 2E–G). However, the secondary structure of the Pekin duck, as shown in the tertiary structure (Figure 2D), has five shortened α-helices and an additional anti-parallel β3-β4, which was located in codons 195–197 and 200–202 (Figure 2H). As a result, it was confirmed that the distribution of the β-sheet structure in the structural model of Pekin duck was increased (8.82%) compared to that in the structural model of chickens, mallards and geese. Detailed information on the tertiary and secondary structure of avian PrPs is described in Table 1.

### 3.4. Evaluation of the Aggregation Propensities in Avian PrPs

**Figure 3:**
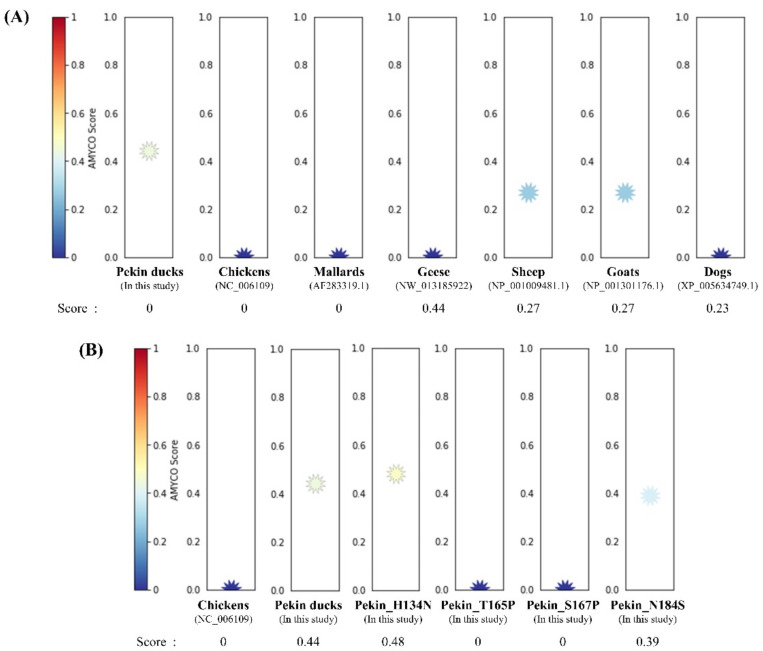
Prediction of the aggregation propensities in avian prion proteins (PrPs). (**A**) The aggregation propensities into amyloid structures in the PrPs were predicted by AMYCO. The text in parentheses indicates protein IDs of the reference sequences used for AMYCO analyses. (**B**) The aggregation propensities according to the substitution with chicken-specific amino acids in the Pekin duck PrP using AMYCO. Pekin_H134N indicates the AMYCO result according to the substitution of His at codon 134 to Asn in the Pekin duck PrP. Pekin_T165P indicates the AMYCO result according to the substitution of Thr at codon 165 to Pro in the Pekin duck PrP. Pekin_S167P indicates the AMYCO result according to the substitution of Ser at codon 167 to Pro in the Pekin duck PrP. Pekin_N184S indicates the AMYCO result according to the substitution of Asn at codon 184 to Ser in the Pekin duck PrP.

To investigate the aggregation propensity into amyloid structures in avian PrPs, including chickens, mallards, geese and Pekin ducks, we investigated the aggregation propensity into amyloid structures in mammalian PrPs, including prion diseases susceptible animals (sheep and goats) and prion diseases resistant animal (dog) using AMYCO. Notably, the Pekin duck PrP was predicted to be the most aggregation-prone PrP among avian PrPs, with a score of 0.44 (Figure 3A). The sheep and goat PrPs with a score of 0.27 were predicted to have more aggregation propensity than dog PrP with a score of 0.23. Except for the Pekin duck PrP, the other avian PrPs had scores of 0.0. In previous studies, chickens were assumed to be resistant to prion diseases; therefore, we measured the effect of substitution in Pekin duck PrP with chicken-specific amino acids using AMYCO. Comparing the chicken PrP sequence as a reference sequence, there are 28 different residues in the Pekin duck PrP, including codons 6, 9, 11, 16, 38, 54, 82, 91, 94, 134, 147, 163, 165, 167, 184, 193, 197, 198, 199, 205, 209, 234, 240, 244, 249 and 254. Detailed substitution information and prediction scores of the Pekin duck PrP according to the substitution of different residues compared with the chicken PrP sequence are described in Table 2. Of the 28 substitutions, 4 substitutions, namely, His134Asn, Thr165Pro, Ser167Pro and Asn184Ser, showed a change in the AMYCO score according to the substitution with chicken-specific amino acids (Figure 3B). Compared to the score of the wild-type Pekin duck PrP (0.44), the Pekin duck PrP sequence substituted with His134Asn showed an increased score of 0.48, and the substitution of Asn184Ser showed a decreased score of 0.39. Interestingly, Pekin duck PrP sequences substituted with Thr165Pro and Ser167Pro showed a decreased score of 0.0, which is identical to the score of the chicken PrP sequence. Contrary to expectations, there was no change in the score with the insertion of 17 amino acids (GYPQNPGYPHNPGYPGW) between codons 80 and 81 (Pekin duck numbering) within the tandem repeat region and with the insertion of one amino acid (Leu) between codons 248 and 249 (Pekin duck numbering) near the stop codon. The remaining residues showed the same score (0.44) as that for the wild-type Pekin duck PrP sequence.

## 4. Discussion

In this study, we first reported the nucleotide sequence of the *PRNP* gene and amino acid sequence of PrP in the domestic Pekin duck. Since Pekin ducks and mallards (wild ducks) are outwardly similar, we expected that the *PRNP* DNA and PrP amino acid sequences of domestic Pekin ducks and mallards would show high homology. However, unlike our expectation, the PrP amino acid sequence of Pekin duck is highly similar to that of geese (97.3%) rather than that of mallards (82.4%) (Figure 1). In addition, a similar tendency was confirmed in the phylogenetic tree of PrP (Figure S2). Interestingly, the PrP amino acid sequence of the Pekin duck showed a low sequence similarity even with that of the chicken (82.7%) (Figure 1). Since previous studies have reported that chickens showed perfect resistance against prion disease, we postulated that the properties related to the resistance of prion disease would also differ between chickens and Pekin ducks because of the low sequence similarity between these two species. Thus, we investigated whether the difference affects the PrP structure of Pekin ducks through tertiary structure analysis of prion proteins. Interestingly, the Pekin duck PrP showed additional β-sheet structures compared to the chicken PrP (Figure 2). Since PrP^Sc^ has been characterized by a conformational transition into a β-sheet-rich structure [12], Pekin duck PrP, which has a higher proportion of β-sheets, is noteworthy [25,26,33]. In previous studies, researchers have suggested that a change in the α-helices structure may give rise to a proportion of the β-sheet structure [34,35,36]. However, it remains controversial which domain is involved in the conversion into the β-sheet structure; therefore, it is believed that our analysis of the Pekin duck PrP will be helpful for further research.

As shown in Figure 3, we found a higher aggregation propensity of the Pekin duck *PRNP* sequence compared to the chicken *PRNP* sequence. In addition, we observed that substitutions with two chicken-specific amino acids on Pekin duck PrP at codons 165 and 167 dramatically reduced the reduction of amyloid propensity. Interestingly, it was found that the amino acid at codon 165 of the Pekin duck PrP (Thr165) is a Pekin duck-specific amino acid, even compared with the PrP sequences of ten species. Furthermore, two amino acids at codons 165 and 167 are located on the β2-α2 loop on the Pekin duck PrP. In previous studies, the β2–α2 loop within PrP has received attention as a potential property associated with PrP conversion or aggregation, resulting in an impact on protein stability due to structural differences of PrP in prion disease-resistant species [20,21,23]. Moreover, previous studies suggested that the β2–α2 loop plays a pivotal role in an interspecies barrier on the transmission of prion disease. The cross-species barrier of prion disease can be overcome by the substitution of host species-specific amino acids [22,37,38,39]. We also investigated the impact of Thr165Pro and Ser167Pro, however, we did not find a significant structural change in secondary and tertiary structure modeling (data not shown). Since these analyses were performed by in silico analysis, there was a limitation to the accuracy of the data. Thus, further confirmation of actual amyloid propensity using in vitro experiments is highly desirable in the future. Furthermore, studies regarding the influence of Pekin duck-specific amino acids on PrP stability or prion transmission are needed in the future. In addition, since Pekin ducks are classified into two distinct breeds, American and German breeds [29,30], the comparative analysis of the *PRNP* gene between these two breeds is valuable in future study. Furthermore, the DNA sequences of the *PRNP* gene in the other domestic ducks, including Korean native ducks, Longsheng Cui, and Jinding, have not been reported thus far. It is elusive whether the variations seen in Pekin ducks are specific to that breed or are common amongst domestic ducks. Thus, further study of the *PRNP* gene in other domestic breeds is highly desirable in the future. In addition, since this study has been performed with samples from four individual animals, there is a question of whether these sequences can represent all genetic characteristics of Pekin ducks found in this study. Thus, further analysis is needed in large samples of the Pekin ducks.

## 5. Conclusions

We first reported the DNA sequence of the *PRNP* gene in domestic ducks (Pekin ducks). We also found that the Pekin duck PrP sequence is more similar to that of geese than to that of chickens and mallards (wild ducks). Interestingly, the Pekin duck PrP showed a high proportion of β-sheets compared to the chicken PrP, which showed perfect resistance against prion diseases. Notably, Pekin duck PrP also showed an extraordinarily high amyloid propensity. In addition, the Pekin duck PrP with substitutions of chicken-specific amino acids, reduced the amyloid propensity. To the best of our knowledge, this is the first report of the *PRNP* gene in Pekin ducks.

## Figures and Tables

**Table 1:** Detailed information on the 3D model and secondary structure of avian prion proteins (PrPs).

Species	Template	Length (Range)	Distribution of α-Helices		Distribution of β-Sheets	
			Location	Percentage	Location	Percentage
Chickens	1U3M	116	151–161, 179–194,	49.57	136–138, 168–170	5.13
		(126–242)	213–240			
Mallards	1U3M.1	114	157–167, 185–200,	47.83	142–144, 174–176	5.22
		(134–248)	219–246			
Geese	1U3M.1	114	140–150, 168–183,	47.83	125–127, 157–159	5.22
		(117–231)	202–229			
Pekin ducks	2lft.1.A	135	140–147, 151, 168,	32.35	125–127, 157–159, 195–197, 200–202	8.82
		(95–230)	172–182, 206–228			

**Table 2:** Prediction of the aggregation propensities in the Pekin duck prion protein (PrP) with substitutions of different residues compared with the chicken PrP.

Sequence	Residues	Pekin Duck-Conserved	Chicken-Specific	Score
Chickens			Wildtype	0
Pekin ducks		Wildtype		0.44
	80_81ins	-	GYPQNPGYPHNPGYPGW	0.44
	6	Valine	Threonine	0.44
	9	Tryptophan	Cysteine	0.44
	11	Valine	Leucine	0.44
	16	Glycine	Alanine	0.44
	38	Threonine	Alanine	0.44
	54	Glutamine	Histidine	0.44
	82	Tryptophan	Glutamine	0.44
	91	Asparagine	Serine	0.44
	94	Histidine	Asparagine	0.44
	134	Histidine	Asparagine	0.48
	147	Asparagine	Serine	0.44
	163	Glycine	Serine	0.44
	165	Threonine	Proline	0
	167	Serine	Proline	0
	184	Asparagine	Serine	0.39
	193	Glycine	Threonine	0.44
	197	Glycine	Valine	0.44
	198	Proline	Alanine	0.44
	199	Glycine	Alanine	0.44
	205	Threonine	Valine	0.44
	209	Threonine	Asparagine	0.44
	234	Arginine	Glutamine	0.44
	240	Alanine	Threonine	0.44
	244	Leucine	Valine	0.44
	248_249ins	-	Leucine	0.44
	249	Alanine	Threonine	0.44
	254	Isoleucine	Methionine	0.44

## Data Availability

All data generated or analyzed during this study are available from the corresponding author on reasonable request.

## Supplementary Materials

The following are available online at https://www.mdpi.com/2073-4425/12/2/193/s1, Figure S1. Comparison of the amino acid (A) and tandem repeat (B) sequences of the prion protein (PrP) in 10 species. The asterisks indicate Pekin duck-specific amino acids identified in this study. Red box indicates dog-specific amino acid, Asp at codon 163, which is related to the resistance of prion disease in the previous study [16,18,19]. The U indicates a hexapeptide repeat units of avian PrPs and are named from U1 to U5. Figure S2. Phylogenetic analysis of the prion protein (PrP) sequences in 10 species.

## Abbreviations

*PRNP*	Prion protein gene
PrP^Sc^	Pathogenic abnormal prion protein
PrP^C^	normal prion protein
BSE	Bovine Spongiform Encephalopathy
FSE	Feline Spongiform Encephalopathy
MEGA	Molecular Evolutionary Genetics Analysis
NCBI	National Center for Biotechnology Information
ORF	Open reading frame
PCR	Polymerase Chain Reaction
GMQE	Global Model Quality Estimation
QMEAN	Qualitative Model Energy ANalysis
PDB	Protein Data Bank
PrP	Prion protein
