# Supplementary figures and images for: The First Report of the Prion Protein Gene (PRNP) Sequence in Pekin Ducks (Anas platyrhynchos domestica): The Potential Prion Disease Susceptibility in Ducks
Source: Genes (Basel). 2021 Jan 28;12(2):193. doi: 10.3390/genes12020193 (PMC7911840; doi:10.3390/genes12020193)

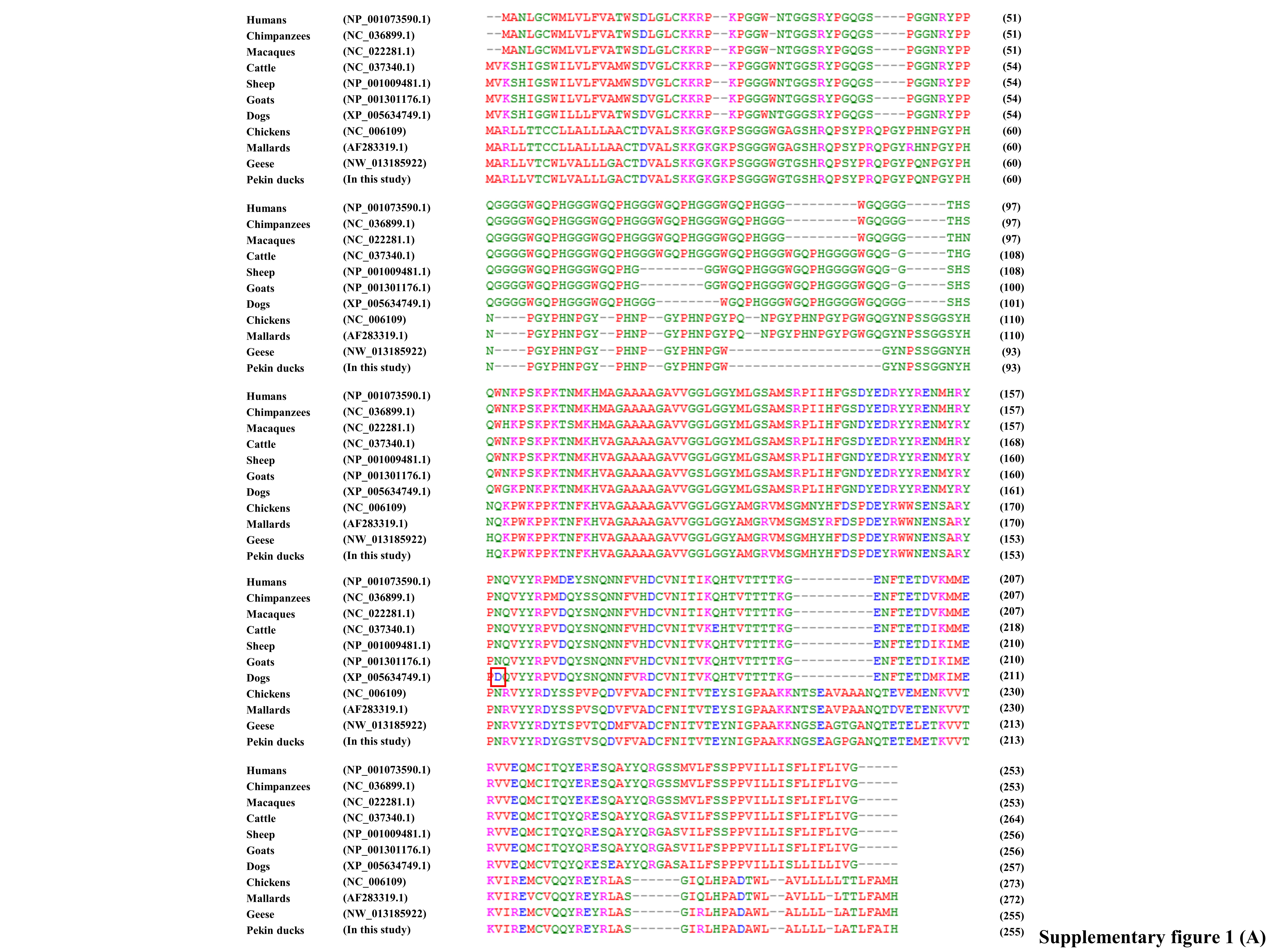

Supplement: Supplementary file 1 [file genes-12-00193-s001.zip › genes-1043946- Supplementary figures/Supplementary figure 1A.JPG]

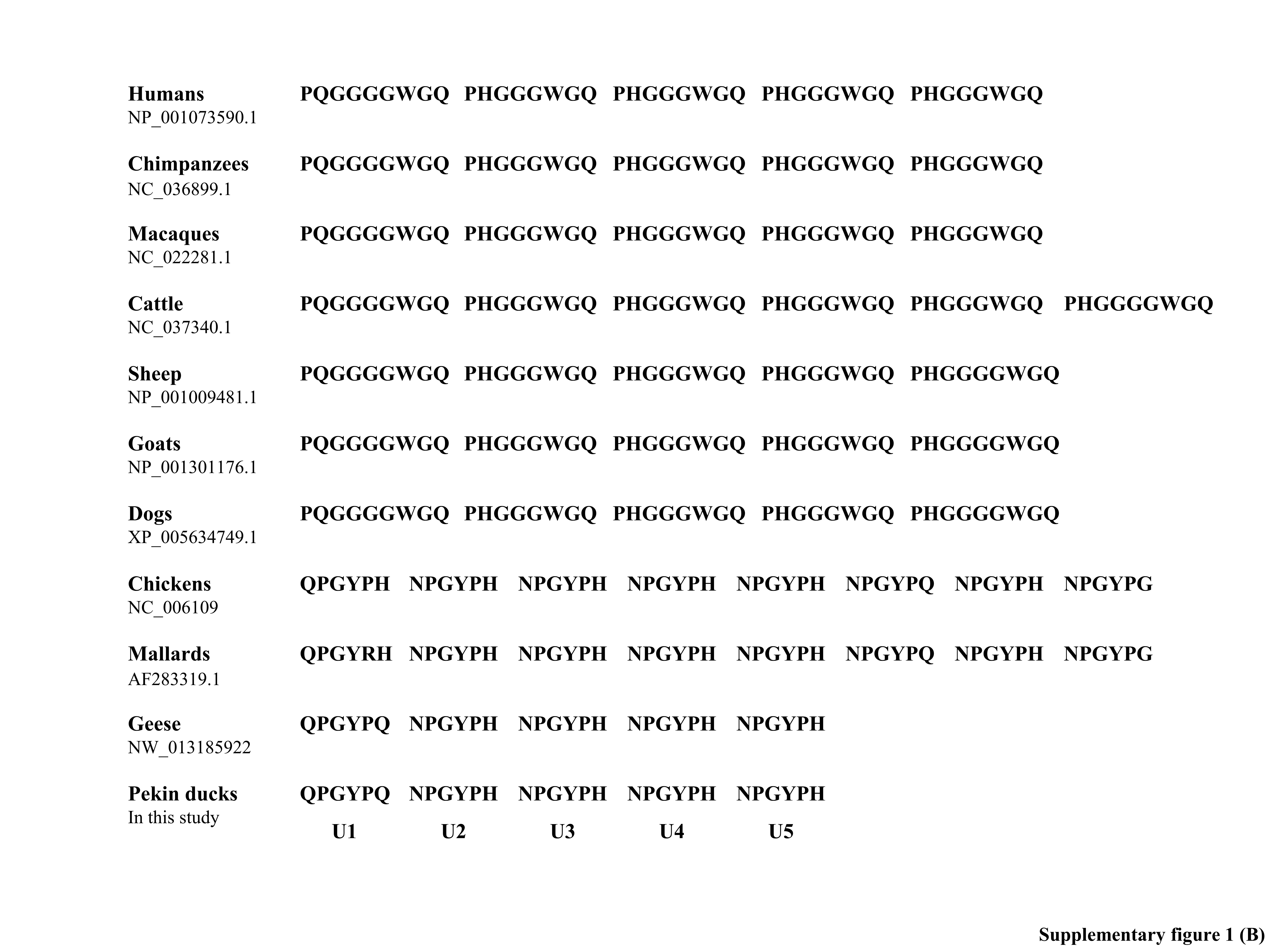

Supplement: Supplementary file 1 [file genes-12-00193-s001.zip › genes-1043946- Supplementary figures/Supplementary figure 1B.JPG]

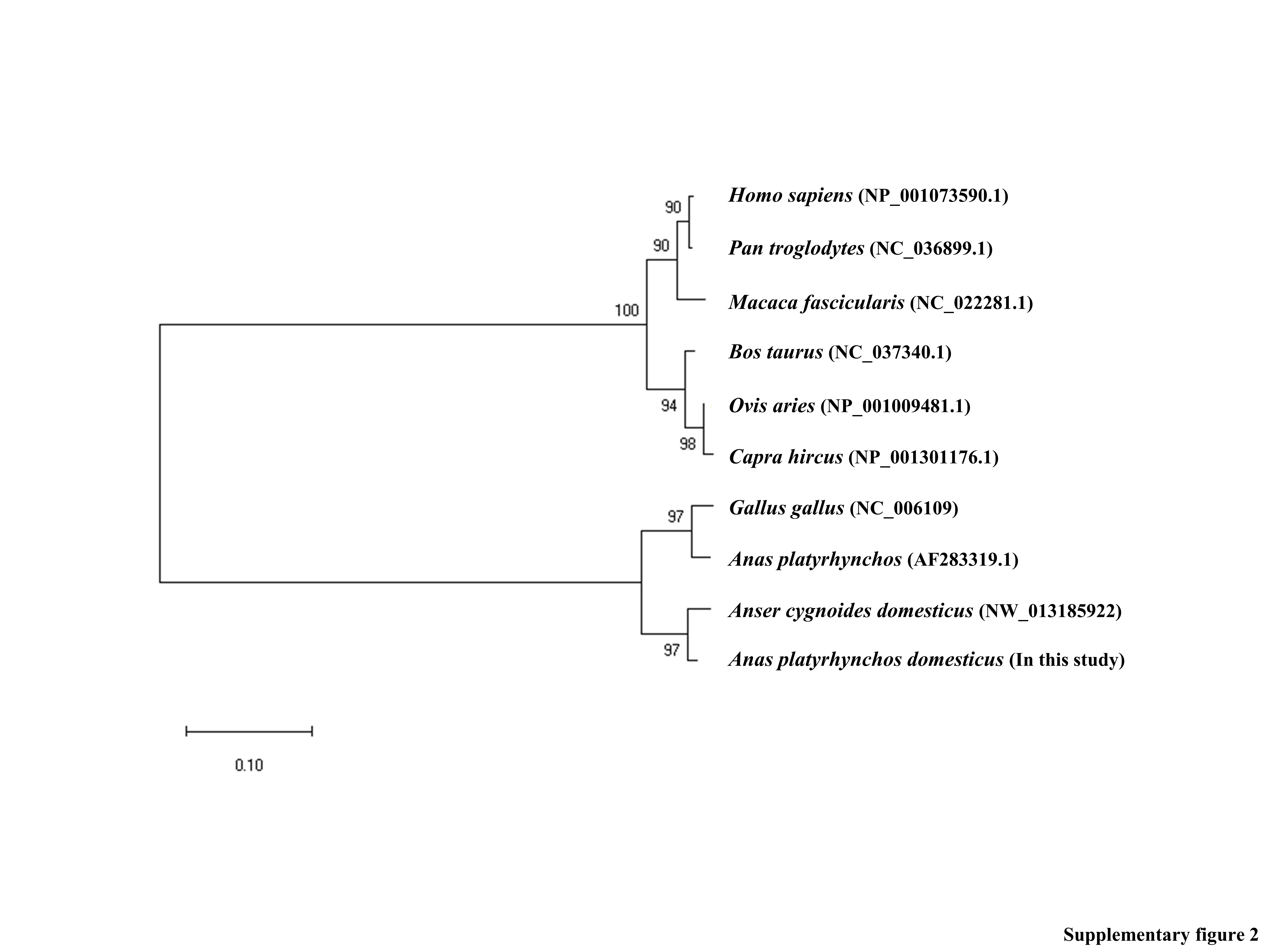

Supplement: Supplementary file 1 [file genes-12-00193-s001.zip › genes-1043946- Supplementary figures/Supplementary figure 2.JPG]
